# Sound production in piranhas is associated with modifications of the spinal locomotor pattern

**DOI:** 10.1242/jeb.242336

**Published:** 2021-05-04

**Authors:** Marine Banse, Boris P. Chagnaud, Alessia Huby, Eric Parmentier, Loïc Kéver

**Affiliations:** 1 Laboratoire de Morphologie Fonctionnelle et Evolutive, Université de Liège, 4000 Liège, Belgium; 2 Department Biology II, Ludwig-Maximilians-University Munich, 82152 Planegg, Germany; 3 Institute for Biology, Karl-Franzens-University Graz, 8010 Graz, Austria

**Keywords:** Activation patterns, Electromyography, Evolution, Locomotion, *Pygocentrus nattereri*, Social communication

## Abstract

In piranhas, sounds are produced through the vibration of the swim bladder wall caused by the contraction of bilateral sonic muscles. Because they are solely innervated by spinal nerves, these muscles likely evolved from the locomotor hypaxial musculature. The transition from a neuromuscular system initially shaped for slow movements (locomotion) to a system that requires a high contraction rate (sound production) was accompanied with major peripheral structural modifications, yet the associated neural adjustments remain to this date unclear. To close this gap, we investigated the activity of both the locomotor and the sonic musculature using electromyography. The comparison between the activation patterns of both systems highlighted modifications of the neural motor pathway: (1) a transition from a bilateral alternating pattern to a synchronous activation pattern, (2) a switch from a slow- to a high-frequency regime, and (3) an increase in the synchrony of motor neuron activation. Furthermore, our results demonstrate that sound features correspond to the activity of the sonic muscles, as both the variation patterns of periods and amplitudes of sounds highly correspond to those seen in the sonic muscle electromyograms (EMG_sonic_). Assuming that the premotor network for sound production in piranhas is of spinal origin, our results show that the neural circuit associated with spinal motor neurons transitioned from the slow alternating pattern originally used for locomotion to a much faster simultaneous activation pattern to generate vocal signals.

## Abstract (precis)

**Summary:** In piranhas, the sound production system used in social communication probably evolved from the spinal locomotor system via modification of the muscles and of the neuronal circuits controlling them.

## INTRODUCTION

The ability to produce sounds for social communication (courtship, spawning, agonistic behavior, competitive feeding, etc.) is widespread across vertebrates, including fishes. It has evolved independently in many phylogenetically distinct fish taxa, hence the existence of several types of sound-producing mechanisms (Fine and Parmentier, 2015). In line with the diversity of sound-production mechanisms comes a variety of neuronal circuit organizations and activation patterns (for review, see Ladich and Bass, 1998; Carlson and Bass, 2000; Ladich and Bass, 2005). While our knowledge about these neuronal circuits is often limited to the location of the motor neurons innervating the sound-producing muscles, with some notable exceptions (Bass et al., 2015), the activation patterns recorded at the muscles or the nerves innervating those muscles allow us to readily identify some shared and divergent properties of the sound-generating circuits.

One of the mechanisms to produce such acoustic signals originates from contractions of paired muscles associated with the swim bladder (Parmentier and Fine, 2016). In piranhas, these sonic muscles insert on transverse enlargements at the base of the second ribs articulating on the third vertebrae and are connected with a common broad tendon that surrounds the swim bladder ventrally (Ladich and Bass, 2005). Piranha sonic muscles are solely innervated by spinal nerves (third, fourth and fifth), meaning that all vocal motor neurons (ca. 150 per vocal muscle) are located in the spinal cord (Ladich and Bass, 2005; Onuki et al., 2006). In many other sonic teleosts, sonic swim bladder muscles are innervated by occipital nerve roots or by a combination of spinal and occipital nerve roots (for review, see Ladich and Bass, 1998, 2005).

The sonic ability in piranhas apparently resulted from an exaptation process (Mélotte et al., 2019), i.e. the development of a new function in structures originally shaped for a different purpose (Parmentier et al., 2017). Sonic muscles in piranhas likely originated from modifications of the locomotor hypaxial musculature (Mélotte et al., 2019) as supported by their innervation pattern, composed of spinal nerves only. These recent findings raise an interesting question: which changes to the sound-producing muscles and their underlying neural circuit are required to transition a system initially involved in slow movements (locomotion) to be able to generate fast movements – a prerequisite for swim bladder sound production (Fine et al., 2001; Fine, 2012)?

To perform high-speed contractions necessary to become sonic, skeletal muscles usually have to undergo a series of modifications. For instance, they may be characterized by: (1) a reduction in fiber diameter (Tavolga, 1964; Fine et al., 1990; Parmentier and Diogo, 2006); (2) a restricted quantity of myofibrils (Rome et al., 1996; Millot and Parmentier, 2014; Parmentier et al., 2021); and/or (3) an increase in sarcoplasmic reticulum volume (Fawcett and Revel, 1961; Revel, 1962; Josephson and Young, 1985; Appelt et al., 1991; Schaeffer et al., 1996; Rome and Lindstedt, 1998; Syme and Josephson, 2002). Many sonic muscles also have an increased proportion of mitochondria (Rome et al., 1996; Schaeffer et al., 1996) if sustained calling performances are required (Millot and Parmentier, 2014; Nelson et al., 2018; Parmentier et al., 2021). The sonic muscles of the red-bellied piranha *Pygocentrus nattereri* show all of the listed adaptative anatomical features (Millot and Parmentier, 2014). By bilaterally stimulating the vocal muscles and simultaneously recording vibrations of the swim bladder wall, Millot et al. (2011) moreover highlighted the ability of sonic muscles to contract at high speed because their contraction rate can reach frequencies (e.g. 150 Hz) higher than the pulse rate of natural ‘bark’ sounds. Furthermore, high-speed sonic muscles could be skeletal muscles that were delayed in their normal development (Millot and Parmentier, 2014).

Modifications relative to the neural circuit and the activation patterns are, however, more enigmatic. The paired sonic muscles contract synchronously in batrachoidids, such as in the plainfin midshipman *Porichthys notatus* (Bass and Baker, 1991), whereas sound production in the sea robin *Prionotus carolinus* is generated by an alternating contraction pattern (Bass and Baker, 1991; Connaughton, 2004). In both systems, the innervation pattern of the sonic muscles originates from occipital nerve roots (Evans, 1973; Bass and Baker, 1991, 1997) and the evolutionary origin of these muscles remains uncertain. In contrast, the origin of vocal muscles has been most likely unraveled in piranhas (Mélotte et al., 2019), but the activation pattern remains to be determined.

As piranha motor neurons are exclusively spinal and thus most likely evolved directly from locomotor motor neurons, one would expect a typical alternate activation of the left and right sonic muscles as shown for the sea robin, for instance (e.g. Bass and Baker, 1991). Kastberger (1981b) challenged this postulate by showing that electrical stimulation of the piranha medulla induced synchronous contractions of the left and right sonic muscles in *Serrasalmus serrulatus*. He suggested the existence of strong electrical coupling of the motor neurons in the vocal system of piranhas, similar to the one seen in toadfishes (Bass and Baker, 1990; Bass et al., 1994), which could, however, not be experimentally verified in *P. nattereri* and *Serrasalmus* *rhombeus* (Ladich and Bass, 2005)*.* Assuming that the electrical stimulation of the medulla has not resulted in an artificial activation pattern in *S. serrulatus*, the simultaneous activation of vocal muscles in this species suggests either that simultaneous contractions of the sonic muscles evolved through different neural paths in piranhas and midshipman, or that *P. nattereri* and *S. rhombeus*, but not *S. serrulatus*, have retained the primitive activation pattern of fish locomotor muscles.

The objective of this study was to establish whether the contractions of the left and right sonic muscles in *P. nattereri* are synchronous or alternating as would be expected from a locomotor-derived spinal central pattern generator in carangiform swimming fishes. Using electromyography, we recorded for the first time the activation patterns of both sonic muscles during voluntary sound production in piranhas as well as the activity of locomotor muscles during swimming. We took advantage of these data to investigate the link between the activation pattern of the sonic muscles and the acoustic features of the ‘bark’ sound (i.e. signal duration, number of pulses, variation in periods, amplitudes and latencies). Assuming that the premotor network for sound production in piranhas is of spinal origin, our results suggest that part of the neural circuit associated with spinal motor neurons transitioned from the typical slow alternating pattern originally used for locomotion to a much faster simultaneous activation pattern to generate vocal signals.

## MATERIALS AND METHODS

### Animals

Eight specimens of the red-bellied piranha (*Pygocentrus nattereri*, Kner 1858) [135–178 mm standard length (SL)] were purchased from ‘Les aquariums de Marbais’ (Belgium) and housed in the Laboratory of Functional and Evolutionary Morphology (Liège University, Belgium). As sexual dimorphism has never been reported in their sound-producing apparatus and all individuals produce similar ‘bark’ sounds when hand-held, sex was not determined. Fish were maintained in an 840 l freshwater tank at 26°C (12 h:12 h light:dark cycle) and fed three times a week with frozen mussels. All procedures were approved by the ethical commission of Liège University (protocol 2110).

### Electromyography

The red-bellied piranha produces at least three types of sounds with two different mechanisms (Millot et al., 2011). Whereas ‘types 1 and 2’ sounds are produced by rapid contractions of sound-producing muscles, ‘type 3’ sounds are produced by a rapid snapping of the jaws. We focused here on this first sound type, also called ‘bark’ sound, composed of several pulses (Millot et al., 2011). This sound is mostly produced during frontal display between two individuals in free-swimming fish (Millot et al., 2011) and when being hand-held (Markl, 1971; Kastberger, 1981a,b; Mélotte et al., 2016).

EMG recordings were carried out in an 84 l tank with bipolar electrodes fashioned from Synflex enameled copper wires (50 µm outer diameter, 40 µm core diameter; Bervaes & Fils SA, Liège, Belgium), following the method described by Parmentier et al. (2013). Fish were anesthetized with MS-222, removed from water and covered with wet paper tissue to avoid desiccation during the procedure of EMG electrode implantation. Electrode wires were secured to the dorsal fin with a suture and cyanoacrylate glue (histoacryl adhesive glue). Every fish could recover from the anesthesia for ∼15 min before the recordings started. Every fish was tested individually and water from the home tank was used to fill the experimental tank before each trial. Two specimens out of the eight were used in both experiments.

For sonic muscle electromyograms (EMG_sonic_), two electrodes were implanted in the left and right sonic muscles and one electrode was placed as a reference in the epaxial musculature, dorso-caudally to the sonic muscles (i.e. ∼2 cm above and 1 cm behind the tip of the first electrodes). Individuals were gently held in the experimenter hand to stimulate voluntary sound production while simultaneously recording both the emitted sounds and the activity of the muscles. During sound production, we were able to record signals from both sonic muscle electrodes in four out of six specimens. In the other two fish, we obtained signals from one of the two sonic muscle electrodes. After examination of all sounds and EMG_sonic_ signals, we concluded that for these trials, one electrode was not recording, most likely because it was not well inserted into the sonic muscle. Our conclusion is supported by two observations. (1) In our recordings, every sound pulse was preceded by an EMG_sonic_ peak. In case of alternance, this is not the pattern we should have observed but instead one EMG_sonic_ peak every two sound pulses. (2) Sounds generated with one or two sonic muscles are expected to show some differences but sound signals associated with the EMGs recorded for only one or the two sonic muscles were highly similar (see Fig. S1).

For locomotion-related EMGs (i.e. locomotor muscle electromyograms, EMG_loco_), we bilaterally recorded the activity of the anterior and caudal body musculature during freely swimming behavior. Two electrodes were placed into the left and right hypaxial musculature (i.e. just below the lateral line, on a vertical axis at the position of the rostral end of the dorsal fin) and two electrodes into the left and right musculature of the caudal peduncle, as close as possible to the midline. The swimming behavior was induced by generating a water current in the tank (i.e. water flowing through a tube connected to a tank with a higher water level was used to avoid electrical noise generated by pumps). During this experiment, we simultaneously recorded signals from the four electrodes in one individual and signals from the pair of electrodes inserted into the caudal and anterior body musculature in one and two individuals, respectively. The absence of signals from the second pair of electrodes in the latter fishes could be due to: (1) the electrodes not properly recording, as concluded for the EMG_sonic_ signals; or (2) the muscles in one of the two areas not being activated (e.g. some swimming behaviors may only require activation of the muscles in the peduncle). Moreover, some electric noise sometimes prevented the signals from being analyzed. A variety of muscle-activation patterns could be detected in fish (e.g. station holding, orientation behavior, swimming). Here, we only focused on swimming patterns.

The signals from the EMG electrodes were amplified 1000 or 10,000 times based on their intensity, band passed (10−10,000 Hz) and notched filtered (50 Hz) with a differential amplifier (AM Systems model 1700, Sequim, WA, USA). A USB sound card (Motu UltraLite-mk4, Cambridge, MA, USA) digitized the EMG activity at a sampling rate of 44,100 Hz in Adobe Audition 2.0 software (Adobe, San Jose, CA, USA). Sounds were recorded with a hydrophone (sensitivity: −163.7 dB re. 1 V μPa^−1^; HTI-96-Min Series; High tech, MS, USA) connected to a Tascam DR-05 digital audio recorder (Wiesbaden, Germany) and routed to a channel of the USB sound card so that sounds and EMGs were synchronized.

After the EMG experiments, the fish were euthanized with an overdose of MS-222 and the electrode wires were cut 1 cm away from the skin. Electrode location was then checked using dissections or computed tomography. Four fishes were scanned (Scanner Siemens Somaton Sensation 16-slice, Siemens AG, München; maximal resolution of the isotropic voxel: 600 µm) at the Veterinary Institute of the University of Liège. For two specimens, electrodes and morphological structures of interest were reconstructed in AMIRA (version 6.2.0) for illustration purposes.

**Fig. 1.**
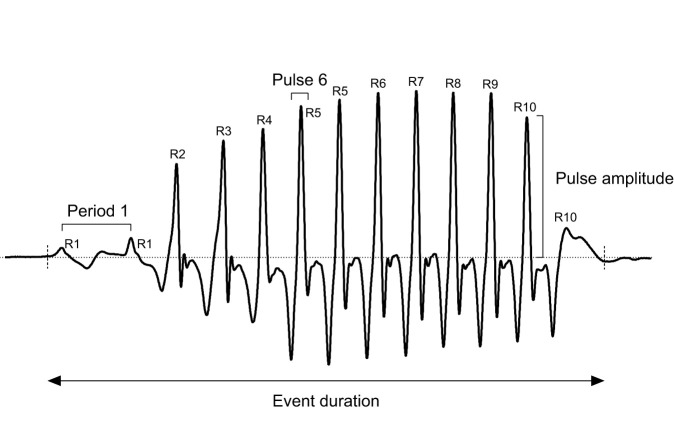
**Variables measured on the waveform of a *Pygocentrus nattereri* sound.** Sound composed of 13 pulses, duration (ms), periods (measured as the peak-to-peak intervals between two consecutive pulses, ms) and pulse amplitudes. Ranks were used to describe amplitude, period and sonic muscle electromyograms (EMG_sonic_)-sound latency modulations within signals. The rank assigned to each pulse of the presented sound is also indicated and varies from R1 to R10. They were assigned to the pulses based on the relative position of each pulse in the sound, using this formula: relative position of a pulse=(absolute position of the pulse – absolute position of first pulse) / (absolute position of the last pulse – absolute position of the first pulse). See Materials and Methods, ‘Statistical analysis’; Table S1.

EMGs and sound recordings were manually investigated using the software Avisoft-SAS Lab Pro 5.2.13 (Avisoft Bioacoustics, Glienicke, Germany). Sixty sounds (‘barks’) recorded from six individuals (10 sounds per individual) and the associated EMG_sonic_ were analyzed. Four variables were measured from the signals (Fig. 1): (1) event duration (defined as the beginning of the first pulse to the end of the last pulse, ms); (2) number of pulses in a signal; (3) pulse periods (measured as the peak-to-peak intervals between two consecutives pulses, ms); and (4) pulse amplitudes (the maximum amplitude of each pulse). The latency between the EMG_sonic_ and the sound was also measured (as the peak-to-peak interval between the EMG_sonic_ pulse or activation potential and the highest peak of the respective pulse in the sound, ms). Sonic muscle synchronization was assessed by measuring the time lag between activation potentials of the left and right EMG_sonic_ signals (*N*_signals_=40, *N*_fish_=4). We also measured the activation rate on both sides of the rostral and/or caudal musculature (*N*_events_=40, 1 s per event, *N*_fish_=4) and the signal-to-noise ratios (SNRs), measured as the differences between the highest peak in EMG_sonic_ or in a 1 s window of EMG_loco_ and the highest peak in a same duration window in the background noise preceding each signal in EMG_sonic_ (*N*_signals_=60, *N*_fish_=6), or in the same swimming event in EMG_loco_ (*N*_events_=40, 1 s per event, *N*_fish_=4). The temporal relationship between the activation of the ipsilateral rostral and caudal locomotor musculature was also measured (*N*_events_=10, 1 s per event, one side).

### Statistical analyses

For all statistical tests, Shapiro–Wilk tests were first used to examine the distribution of the data. Sometimes, normality could be reached using log transformations. When the assumption of normality was met, Levene's tests were performed to assess the assumption of homoscedasticity. This allowed us to decide if parametric or non-parametric tests should be used in the analyses. We first performed an unpaired *t*-test to compare the activation rates of the sonic and locomotor muscles, and an unpaired Welch's *t*-test to compare their SNRs because variances were unequal. In addition to these tests, we performed an analysis of covariance (ANCOVA) to test the effect of the signal type (sound versus EMG_sonic_ signal) and SL on the duration of the signals. We also built statistical models to test whether the selected parameters (signal type, SL and ‘rank’ when appropriate) have an effect on the number of pulses, periods and amplitudes. The choice of each model was based on the type of data to analyze and their distribution. The selected models were those that best fitted the data as well as their respective predictions. For each response variable, the best model required, as independent variables, both the signal type and SL to properly describe the data. The predictor variable ‘rank’, hereafter explained, was also required in the models for periods and amplitudes. It allowed us to investigate the temporal variation patterns of periods and amplitudes within the signals and to compare these patterns between sounds and EMG_sonic_. For the number of pulses, we built a generalized linear model (GLM) using the *glm* function for Poisson family with a log-link function as this model type is the most suitable for count data. The model that fitted the best periods data under repeated muscle contractions within signals was an exponential decay model as used by Morel et al. (2019) and Sheng et al. (2021) to model muscle fatigue (i.e. the decay in maximum force produced by muscles) under repeated contractions. Therefore, we built a Gamma GLM with a log-link function using the *glm* function. The dispersion parameter was fixed to one. Finally, for normalized amplitude (explained below), we ran a second-order polynomial model using the *lm* function. The variable ‘rank’ was used as the second-degree term.

Because signals were composed of 9–17 pulses, sorting the data from the different variables (i.e. period, amplitude, latency) based on the absolute pulse position within an event (i.e. a sound) would have impaired the description of the variation patterns. Therefore, each pulse within a signal was assigned to a rank ranging from 1 to 10 based on its relative position [relative position of a pulse=(absolute position of the pulse–absolute position of first pulse)/(absolute position of the last pulse–absolute position of the first pulse)] (Fig. 1; Table S1). In addition, relative amplitudes of sounds and EMG_sonic_ were normalized to allow the comparison of amplitude modulations between these signal types. First, the ‘amplitude delta’ of each pulse in a signal was calculated by subtracting the amplitude of the smallest pulse in the signal from the amplitude of every other pulse. Second, for each signal type, the ‘amplitude deltas’ were normalized using the formula:
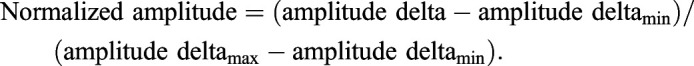


For the comparison between the features of the EMG_sonic_ and the sound waves, the number of pulses, the duration of the signals and the periods were measured on the EMG of one sonic muscle because activation potentials were highly similar and synchronized in both sonic muscles (i.e. a peak in the EMG_sonic_ of one of the two muscles was always synchronous with a peak in the EMG_sonic_ of the muscle on the other side). Amplitudes were, however, averaged from the EMG traces of both sonic muscles as they often differed between the left and right sides.

Using a Kruskal–Wallis test, variation in latencies within the signals was investigated based on the rank they were assigned to. Because we observed variations in periods, amplitudes and latencies within the signals, Dunn's tests for multiple pairwise comparisons were run between ranks for each signal. Alpha levels were adjusted with a Bonferroni correction for multiple testing. All statistical tests were performed in R (version 1.2.1335). Results are presented as means±s.d. Significance level was determined at *P*<0.05.

## RESULTS

### Sonic EMG analysis

**Fig. 2.**
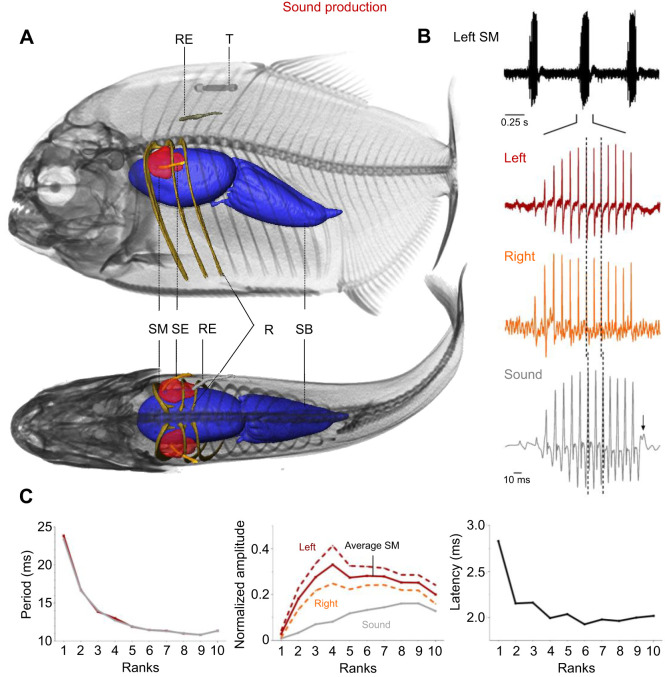
**Activation patterns of the sonic muscles in *Pygocentrus nattereri.*** (A) Left lateral (top) and dorsal (bottom) views of the location of the EMG recording) electrodes and morphological structures of interest during the recording of the sonic muscles. All 3D reconstructions were done using AMIRA (version 6.2.0). R, ribs; RE, reference electrode; SB, swim bladder; SE, sonic muscle electrode; SM, sonic muscle; T, tag name of the individual. (B) EMGs of the sonic muscles (EMG_sonic_) and waveform of the associated sound. The activation potentials of the two sonic muscles are highly synchronized. The vertical dashed bars indicate correlated peaks between sound and EMG_sonic_. The small arrow in the sound trace indicates the additional pulse with respect to the EMG_sonic_. (C) Variation in average period (gray line, sound; red line, sonic muscle), normalized amplitude (gray line, sound; orange and red dashed lines, right and left sonic muscles, respectively; solid red line, average of the two sonic muscles) and latency between sound and EMG_sonic_ pulses. Ranks correspond to the normalized positions of the pulses in the signals.

During sound production, EMG_sonic_ potentials could be detected from both the right and left sonic muscles. Their EMGs (EMG_sonic_) were characterized by short (2.83±1.13 ms, *N*_sounds_=10, *N*_fish_=4) but large amplitude pulses (SNR: 32.23±12.76), suggesting that muscle fibers on each side of the body were activated synchronously (Fig. 2A,B). Both sides were also highly synchronized, as the average time lag between the activation potentials on either side was ∼0.3 ms (0.26±0.28 ms). The contraction frequency of the sonic muscles, calculated as the proportional inverse of the period, reached on average 78.77±18.66 Hz. The EMG_sonic_ activation potentials each preceded a sound pulse (Fig. 2B) that lagged on average 2.15±0.74 ms.

While there is a significant effect of SL on the number of pulses in the signals (Table 1, *P*<0.01), we did not find an effect of the signal type (Table 1, *P*>0.05). Similarly, the results of the ANCOVA showed that the signal type did not affect the duration of the signals (Table 2, *P*>0.05) nor did SL (Table 2, *P*>0.05). These data show that the two signals do not differ in terms of duration (respectively, 180.95±27.10 ms and 173.03±26.82 ms for sounds and EMG_sonic_ signals) nor in their number of pulses (respectively, 13.4±1.82 and 12.72±1.81 for sounds and EMG_sonic_ signals). Note that a smaller pulse associated with no activation potential was frequently observed at the end of the sounds (Fig. 2B, black arrow). Sounds and EMG_sonic_ signals do not differ either (Table 3, *P*>0.05) in their periods (respectively, 13.95±6.11 ms and 14.03±6.28 ms). These results support the hypothesis that each sound pulse results from a simultaneous activation potential in both sonic muscles. Sounds and averaged EMG_sonic_ signals differed in their normalized amplitude (Table 4, *P*<0.0001). Finally, both periods and normalized amplitude vary with SL (Tables 3 and 4; *P*<0.05). We found that with an increasing size, the duration of the signals remains stable while the number of pulses and the normalized amplitude increase and the periods decrease.

**Table 1.**
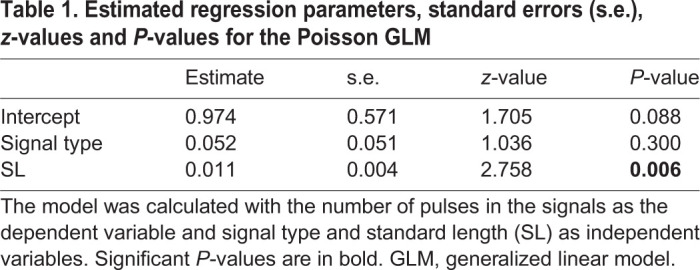
**Estimated regression parameters, standard errors (s.e.), *z*-values and *P*-values for the Poisson GLM**

**Table 2.**
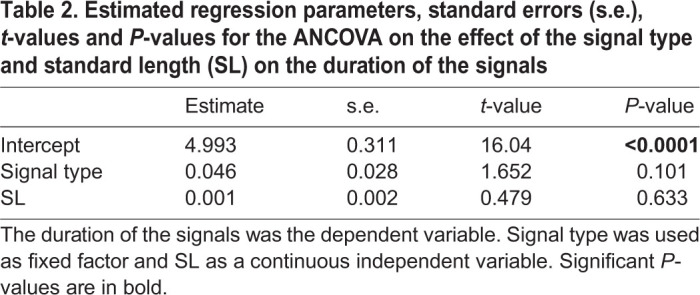
**Estimated regression parameters, standard errors (s.e.), *t*-values and *P*-values for the ANCOVA on the effect of the signal type and standard length (SL) on the duration of the signals**

**Table 3.**
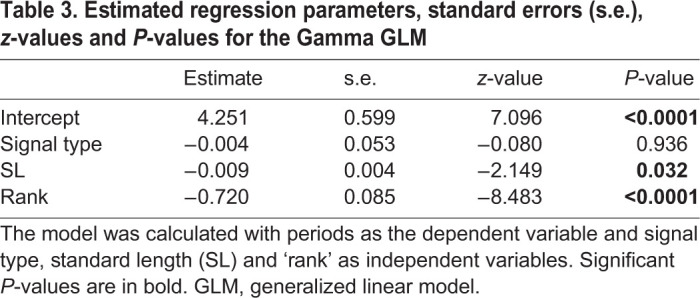
**Estimated regression parameters, standard errors (s.e.), *z*-values and *P*-values for the Gamma GLM**

**Table 4.**
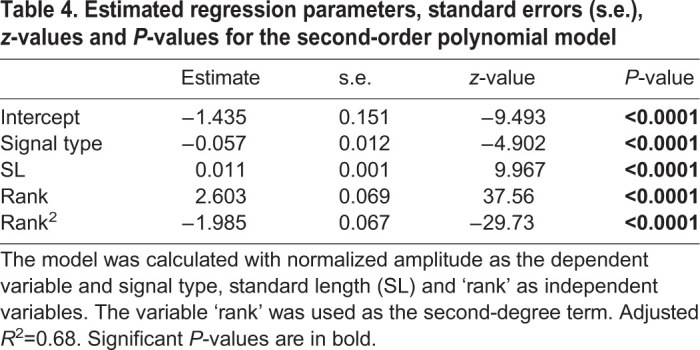
**Estimated regression parameters, standard errors (s.e.), *z*-values and *P*-values for the second-order polynomial model**

Both periods and normalized amplitude also vary along the signals (Tables 3 and 4; *P*<0.0001). The pulse period decreased over the course of the first half of the sound and EMG_sonic_ signals (Fig. 2C). Rank 1 periods were longer than rank 3–10 periods in both sound and EMG_sonic_ signals. Similarly, ranks 2 and 3 periods were, respectively, longer than rank 4–10 periods and rank 5–10 periods in both signals. Finally, ranks 4 and 5 periods were, respectively, longer than rank 6–10 and rank 8–9 periods in both sound and EMG_sonic_ signals (Dunn's multiple comparison tests, *P*<0.05; Table S2). Although normalized amplitude was different between the sounds and averaged EMG_sonic_ signals, a similar pattern of variation was observed within sounds and EMG_sonic_ (Fig. 2C): normalized amplitude increased, reached a plateau and then decreased. Averaged EMG_sonic_ normalized amplitude, however, reached a plateau earlier in the signals compared with sounds. For both the sounds and averaged EMG_sonic_ signals, significance tests showed that rank 1 normalized amplitude was smaller than all the other ranks whereas ranks 2–10 normalized amplitudes did not differ. The only difference between the two types of signals was observed for rank 2 normalized amplitude being, in sound, smaller than rank 10 normalized amplitude (Dunn's multiple comparison tests, *P*<0.01; Table S2). The EMG_sonic_-sound latencies measured between rank 1 pulses were significantly longer than those measured for the other ranks (Fig. 2C; Kruskal–Wallis test, *P*<0.0001; Dunn's multiple comparison tests, *P*<0.0001; Table S2).

### Locomotor EMG analysis

**Fig. 3.**
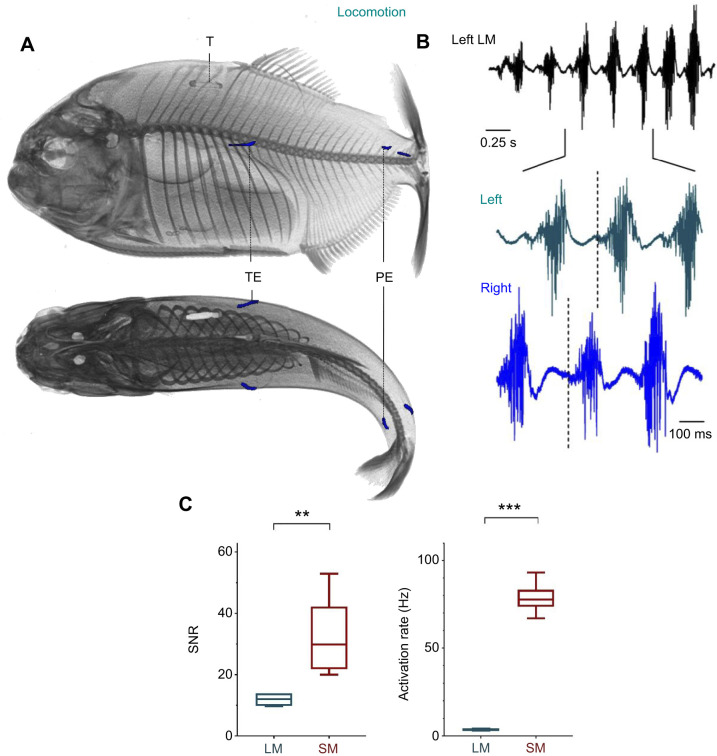
**Activation patterns of the locomotor muscles in *Pygocentrus nattereri.*** (A) Left lateral (top) and dorsal (bottom) views of the location of the EMG recording electrodes and morphological structures of interest during the recording of the locomotor muscles. All 3D reconstructions were done using AMIRA (version 6.2.0). PE, electrode located in the caudal musculature; T, tag name of the individual; TE, electrode located in the rostral hypaxial musculature. (B) EMGs of the hypaxial locomotor musculature (EMG_loco_). The vertical dashed bars indicate the start of correlated activation potentials in the left and right locomotor muscles and highlights their alternate activation pattern. (C) Boxplots representing the signal-to-noise ratio (SNR) and activation rate of the sonic muscles (SM, *n*=4) and hypaxial locomotor muscles (LM, *n*=6). The box boundaries represent the first and third quartiles, the whiskers indicate the minimum and maximum values, and the lines indicate the medians. The asterisks indicate how statistically significant the differences were between the locomotor and sonic muscles: **P≤0.01; ***P<0.001).

Both the rostral and caudal locomotor muscle pairs exhibited an alternate pattern of contraction (i.e. one side of the body contracted at a time) during swimming activity (Fig. 3A,B). The swimming frequency of locomotor muscles varied from 2.5 to 6 Hz (3.77±0.73 Hz, *N*_events_=40, 1 s per event, *N*_fish_=4) and was consistent between the rostral and caudal musculature (*N*_events_=10, 1 s per event, *N*_fish_=1).

In comparison to sonic muscles (78.77±18.66 Hz), the activation rate of locomotor muscles was thus more than 20 times lower (*t*-test, *P*<0.0001) (Fig. 3C). The mean SNR recorded for EMG_­loco_ (11.79±6.59) was also more than 2 times lower than that of the EMG_sonic_ (32.23±12.76) (Welch's *t*-test, *P*=0.01) (Fig. 3C), most likely because EMG traces in the locomotor muscles were composed of asynchronous activation potentials (Fig. 3B). We observed an averaged delay of 63.14±29.31 ms (range: 6–138 ms) between the activation of the ipsilateral rostral and caudal locomotor musculature; a common observation during undulatory activity in the spinal cord (Grillner and Kashin, 1976).

## DISCUSSION

### Locomotor circuits as ancestors of vocal circuits

In most fishes using trunk and caudal fin movements for undulatory propulsion, swimming is generated by alternating contractions of the locomotor muscles on both sides of the body (Grillner and Kashin, 1976) induced by spinal locomotor networks (Kiehn, 2016). Our results show that red-bellied piranhas also employ an alternating pattern for the generation of locomotor activity, as expected for carangiform swimming.

Although sonic muscles in piranhas likely originated from hypaxial locomotor muscles (Mélotte et al., 2019) and are also innervated by spinal nerves (Ladich and Bass, 2005; Onuki et al., 2006), our results demonstrate that these muscles involved in sound production are synchronously activated. How did a locomotor system get transformed into a sonic system? Three major modifications between the locomotor and the sonic systems became apparent from our experiments: (1) a transition from a bilateral alternating to a synchronous activation pattern; (2) a switch from a slow- to a high-frequency regime; and (3) an increase in the synchrony of motor neuron activation, as shown by the large and short activation potentials in the EMGs of the sonic muscles.

These modifications directly relate to the different requirements for locomotion and acoustic communication. Whereas EMG_loco_ are mostly characterized by broad activation potentials, suggesting asynchronous activations of the fibers within each locomotor muscle, the EMGs of the sonic muscles are characterized by short but large activation potentials, which infer synchronous fiber activation within each muscle; a feature detected in other fishes and rattlesnakes (e.g. Cohen and Winn, 1967; Schaeffer et al., 1996). This transition from activation patterns allowing strength modulations (which are necessary for proper locomotion) to a precise synchronous activation pattern of sonic muscle fibers is a prerequisite to generate high-frequency sound pulses, as the muscle activation times must be short. Synchronous fiber activations within each sonic muscle, together with the simultaneous bilateral muscle contraction, likely increase the strength of the sonic muscle contractions, the amplitude of displacement of the swim bladder wall and consequently the uniform compression of the swim bladder. These features allow piranha sonic muscles to generate sounds with adequate amplitude and propagation distance despite a lower proportion of myofibrils than that of the epaxial locomotor musculature (see Millot and Parmentier, 2014 for muscle fiber histology). Superfast sonic muscles and associated spinal motor neurons of piranhas probably evolved under multiple constraints. Here, we present three hypotheses that could explain the reason for this shift from a slow- to a high-frequency regime. (1) Best hearing sensitivity in piranhas is between 50 and 900 Hz (Mélotte et al., 2018). The shift towards the generation of higher-frequency signals could be the result of natural selection for individuals producing more audible signals. (2) The shallow water habitats (i.e. freshwater rivers and streams) of piranhas have high attenuation rates for low frequencies (Rogers and Cox, 1988). On rocky bottoms, the cut-off frequency (i.e. the lowest frequency that can propagate in a specific aquatic environment) decreases from ∼300 Hz in 1 m water depth to ∼30 Hz in 10 m water depth (Rogers and Cox, 1988). Therefore, the production of higher-frequency sounds in piranhas could also be the result of selection for signals with larger propagation distances. (3) Finally, Fine et al. (2001) showed that the electrically stimulated sonic muscles of the toadfish *Opsanus tau* produce maximal sound amplitude and swim bladder wall displacement at frequencies in the vicinity of the fundamental frequency of its boatwhistle (i.e. 150–270 Hz). However, they also found that, despite a significant drop in swim bladder wall displacements for muscle stimulations over 150 Hz, the swim bladder actually becomes more efficient (i.e. larger sound amplitude/swim bladder velocity ratios) at higher frequencies (optimum: 400 Hz). In the red-bellied piranha, Millot et al. (2011) showed that the displacements of the swim bladder wall dropped when sonic muscle stimulations increase from 1 to 150 Hz, yet the loud ‘bark’ sounds are produced at ∼120 Hz. As it was suggested for toadfish (Fine et al., 2001), the piranha swim bladder may be more efficient at high frequency. Therefore, individuals with faster sonic muscles could have been selected because their sound-producing system could be more efficient. These hypotheses are not mutually exclusive.

Such adjustments (i.e. increased frequency, permanent synchronous activation of muscle fibers) surely required the reorganization of a part of the ancestral locomotor circuit (motor neurons and interneurons) to become sonic. Observations in both vertebrates and invertebrates (Katz, 2016) showed that small genetic changes (e.g. gene alteration or suppression) can induce rewiring responsible for alteration of motor behaviors. In mice, for instance, a modification of the neural organization of interneurons (i.e. spinal neurons crossing the midline instead of remaining ipsilateral) in spinal circuits caused a shift from an alternating to a synchronous (i.e. hopping) gait (Kullander et al., 2003). Similar changes might have also occurred in piranhas.

### Vocal neural circuit organization across fishes

**Fig. 4.**
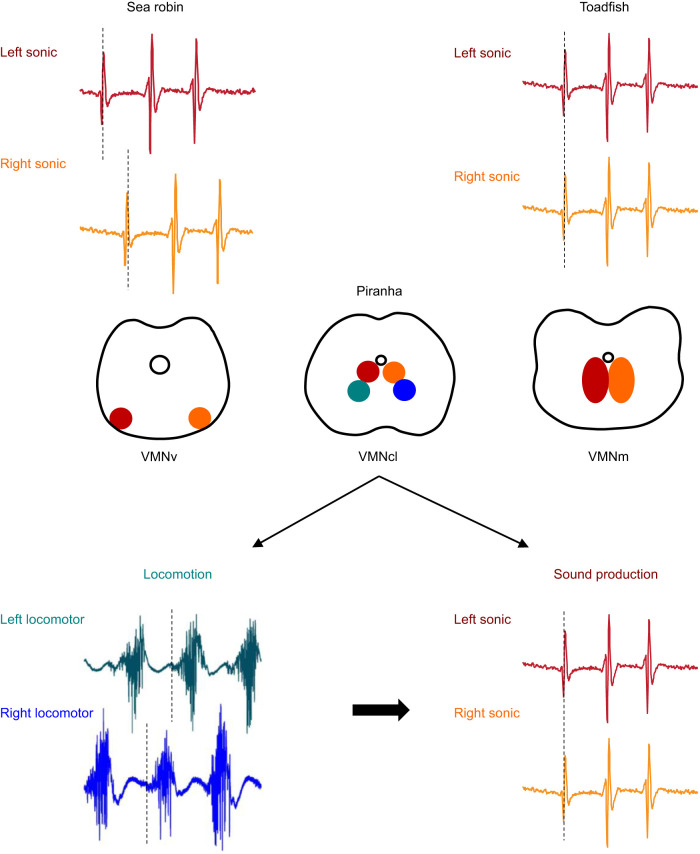
**Schematic representation of the vocal motor neurons location in the hindbrain and spinal cord of the midshipman fish *P. notatus*, the red-bellied piranha *P. nattereri* and the sea robin *P. carolinus* and of the activation patterns of the associated muscles.** The neural organization of the vocal motor neurons of the three species and the activation patterns of the sonic muscles of *P. notatus* and *P. carolinus* are based on Bass and Baker (1991) and Ladich and Bass (2005). The location of locomotor motor neurons of *P. nattereri* and the activation patterns of the associated muscles are also depicted. In the drawings of the spinal cord, colors indicate motor neurons of the left (red) and right (orange) sonic muscles and of the left (green) and right (blue) locomotor muscles. The same colors were used for the EMGs. The large black arrow indicates the transition from the alternate pattern of activity in the locomotor pathway in *P. nattereri* to the synchronous pattern of activity used in acoustic communication. VMN, vocal motor nuclei; v, ventral; cl, centro-lateral; m, midline.

Synchronous contraction of the sonic muscles has been observed in many of the fish species studied, such as for the toadfishes *P. notatus* (Cohen and Winn, 1967) and *O. tau* (Tower, 1908; Skoglund, 1961; Elemans et al., 2014), the weakfish *Cynoscion regalis* (Connaughton et al., 2000) and the pigfish *Congiopodus leucopaecilus* (Packard, 1960). In toadfishes, the neural circuit associated with the paired sonic muscles is characterized by a large midline vocal motor nucleus (VMN) divided in a left and right pool of sonic motor neurons (each one innervating their respective ipsilateral muscle) whose dendrites extend bilaterally (Fig. 4; Bass et al., 1994). Such an organization likely facilitates a concurrent activation of both muscles, as motor neurons of either population share the input of their premotor neurons (Bass and Baker, 1990; Chagnaud et al., 2011). Unlike toadfishes, the activation pattern of the sea robin *P. carolinus* is alternate (Connaughton, 2004) and the motor neurons innervating the right and left sonic muscles are largely separated on each ventrolateral side of the spinal cord (Fig. 4; Bass and Baker, 1991), which, strictly on anatomical terms, does not facilitate a simultaneous activation. As for the sea robin, the dendrites of the sonic motor neurons in *P. nattereri* do not extend bilaterally (Fig. 4; Ladich and Bass, 2005; Onuki et al., 2006). Vocal motor neurons in piranhas thus represent a mix of both conditions, as the neurons are located in two separate but centro-lateral-oriented VMN, each of them innervating the sonic muscle on one side of the body (Fig. 4; Ladich and Bass, 2005; Onuki et al., 2006). Furthermore, the anatomical organization of the sound-producing mechanism in a pair of extrinsic sonic muscles located on each side of the swim bladder and connected with a broad tendon surrounding the swim bladder ventrally in piranhas (Ladich and Bass, 2005) supports the synchronous contraction of these muscles. Indeed, one would expect this system to be more efficient if, as shown here, both muscles contract simultaneously to evenly pressure the swim bladder to produce sounds.

The role of premotor neurons in maintaining synchrony between the two VMN and therefore between the sonic muscles on opposite sides of the body has been supported by studies on batrachoidids (Bass and Baker, 1990; Bass et al., 1994). In *P. notatus*, the synchronous contraction of the sonic muscles is ensured by an electrical coupling of vocal motor neurons through bilateral inputs from premotor neurons adjacent to the VMN (Bass and Baker, 1990; Bass et al., 1994). In *P. nattereri*, the lack of apparent electrical coupling between motor neurons suggests that the bilateral synchronization of the sonic muscles is ensured by chemical coupling only via shared premotor neuronal inputs to sonic motor neurons. In the lamprey, glutamatergic neurons whose axons cross the midline (commissural neurons) are thought to play a role in promoting left–right synchrony (Grillner, 2003). Similarly, left–right alternation in young tadpoles and adult lampreys is thought to be organized by inhibitory glycinergic commissural neurons (Grillner, 2003; Roberts et al., 2008). Transitions of bilaterally alternating to simultaneously contracting patterns readily occur in spinal systems, such as in developing *Xenopus* tadpoles, that modify their locomotor style from undulating to kicking (Beyeler et al., 2008).

### Comparison between sound features and sonic activity

Sound features reflected the activity of the sonic muscles, as sound duration, the number of pulses and pulse periods varied according to the duration, number of pulses and pulse periods of EMG_sonic_ signals. In both sounds and EMG_sonic_, pulse period decreased over the course of the first half of the signals. Amplitudes of the first ranks were also smaller than those of the successive ranks. These results are in agreement with other studies on piranhas (Mélotte et al., 2016) and toadfishes (Fine et al., 2001) where the first sound pulses were characterized by longer periods and smaller amplitudes than the following pulses. Furthermore, we found that the first rank EMG_sonic_-sound latency was longer than the following latencies. Fine et al. (2001) proposed that the longer duration and lower amplitude of the first activation potentials could be due to the initial timing in the release and reuptake of calcium from the sarcoplasmic reticulum during the first contractions cycles. Electrophysiological recordings from vocal motor neurons in midshipman fish suggest that synchronous activity in the motor neuron pool driving the sonic muscles, essential in the aforementioned timing, must first be established (Chagnaud et al., 2012).

In the present study, normalized amplitude was different between the sounds and the averaged EMG_sonic_ but the variation pattern within the signals was similar: amplitude increased, reached a plateau and then decreased. This interesting observation suggests that the number of vocal muscle fibers synchronously activated varies during the sound's emission. Indeed, the small activation potentials and pulse amplitudes recorded at the beginning of a sound may indicate that only few motor neurons are activated at this stage. In the middle portion of the sound, the number of activated motor neurons would then gradually increase until a majority of them are activated, which correlates to the plateau in the EMG_sonic_ pulse amplitude. Such an activation mechanism can be explained by the differential recruitment of motor neurons according to the size principle (Henneman and Mendell, 1981), and as previously suggested for the toadfish vocal system (Chagnaud et al., 2012). The size principle predicts that smaller motor neurons are activated before larger ones due to their higher input resistance (increased excitability). For comparable excitatory synaptic currents, they thus tend to depolarize more and fire earlier than larger motor neurons (Henneman and Mendell, 1981). As vocal motor neurons in piranhas show a range of sizes (Onuki et al., 2006), this assumption seems appropriate.

The fast decrease in vibrations of the swim bladder wall following the stop of electrical stimulation of the sonic muscles in *P. nattereri* testifies the highly damped structure of the swim bladder in piranhas (Millot et al., 2011). However, the small additional pulse observed at the end of most sounds with respect to the EMG_sonic_ activity probably corresponds to the expansion of the swim bladder that is returning to its original position after complete relaxation of the sonic muscles (Fine et al., 2001).

### Conclusion

How do novel behaviors arise from pre-existing substrates? Our comparison between sonic and locomotor activation patterns highlights the modifications of the neural motor pathway that accompanied the suggested and highly supported exaptation process enabling sound production from originally hypaxial locomotor muscles in piranhas. Due to the spinal origin of piranha motor neurons and the most probable shared ancestry of their sonic muscle, piranhas make an ideal model to study evolutionary transitions of motor behaviors, here locomotor to sonic. Future studies should aim at determining the changes undergone at the premotor circuit level and the accompanying changes in intrinsic properties of the neurons within these two different networks.

## Supplementary Material

Supplementary information
