# Supplementary material for: Sound production in piranhas is associated with modifications of the spinal locomotor pattern
Source: J Exp Biol. 2021 May 4;224(9):jeb242336. doi: 10.1242/jeb.242336 (PMC8126449; doi:10.1242/jeb.242336)
Supplement: Supplementary information [file jexbio-224-242336-s1.pdf]

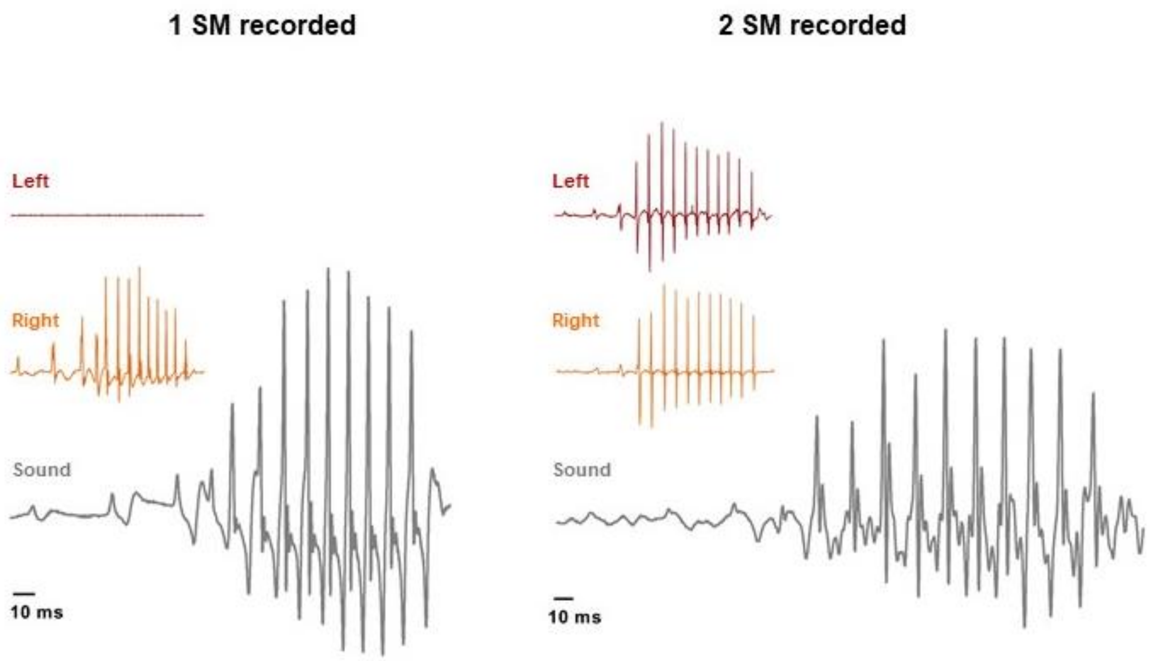

Figure S1. Comparison between the sound waveform obtained when only the EMG of one sonic muscle (SM) was recorded and the sound waveform obtained when the EMGs of the two SM were recorded in *Pygocentrus nattereri*. Note that the two sound waveforms are very similar. Grey line, sound; orange and red lines, right and left sonic muscles, respectively.

Journal of Experimental Biology • Supplementary information

Table S1. Example of the ranking procedure (rank 1 to 10) for the 13 pulses of the sound in Fig. 1.

Absolute position of each pulse in the sound	Relative position of each pulse	Range	Rank
1	0	]-0.001,0.1]	1
2	0.08	]-0.001,0.1]	1
3	0.17	]0.1,0.2]	2
4	0.25	]0.2,0.3]	3
5	0.33	]0.3,0.4]	4
6	0.42	]0.4,0.5]	5
7	0.5	]0.4,0.5]	5
8	0.58	]0.5,0.6]	6
9	0.67	]0.6,0.7]	7
10	0.75	]0.7,0.8]	8
11	0.83	]0.8,0.9]	9
12	0.92	]0.9,1]	10
13	1	]0.9,1]	10

Relative position of a pulse = (absolute position of the pulse - absolute position of first pulse) / (absolute position of the last pulse - absolute position of the first pulse)

Table S2. Comparisons between periods, normalized amplitudes and latencies within sounds and (averaged) EMG_{sonic} signals based on the rank they were assigned to.

Rank periods (sound)	1	2	3	4	5	6	7	8	9
2	NS	-	-	-	-	-	-	-	-
3	**<0.0001**	NS	-	-	-	-	-	-	-
4	**<0.0001**	**0.0029**	NS	-	-	-	-	-	-
5	**<0.0001**	**<0.0001**	**0.0027**	NS	-	-	-	-	-
6	**<0.0001**	**<0.0001**	**<0.0001**	**0.0314**	NS	-	-	-	-
7	**<0.0001**	**<0.0001**	**<0.0001**	**0.0007**	NS	NS	-	-	-
8	**<0.0001**	**<0.0001**	**<0.0001**	**<0.0001**	**0.0064**	NS	NS	-	-
9	**<0.0001**	**<0.0001**	**<0.0001**	**<0.0001**	**0.0002**	NS	NS	NS	-
10	**<0.0001**	**<0.0001**	**<0.0001**	**0.0003**	NS	NS	NS	NS	NS
Rank periods (EMG_{sonic})	1	2	3	4	5	6	7	8	9
2	NS	-	-	-	-	-	-	-	-
3	**<0.0001**	NS	-	-	-	-	-	-	-
4	**<0.0001**	**0.0263**	NS	-	-	-	-	-	-
5	**<0.0001**	**<0.0001**	**0.0059**	NS	-	-	-	-	-
6	**<0.0001**	**<0.0001**	**<0.0001**	**0.0082**	NS	-	-	-	-
7	**<0.0001**	**<0.0001**	**<0.0001**	**0.0006**	NS	NS	-	-	-
8	**<0.0001**	**<0.0001**	**<0.0001**	**<0.0001**	**0.029**	NS	NS	-	-
9	**<0.0001**	**<0.0001**	**<0.0001**	**<0.0001**	**0.0029**	NS	NS	NS	-
10	**<0.0001**	**<0.0001**	**<0.0001**	**<0.0001**	NS	NS	NS	NS	NS
Rank normalized amplitudes (sound)	1	2	3	4	5	6	7	8	9
2	**0.0066**	-	-	-	-	-	-	-	-
3	**<0.0001**	NS	-	-	-	-	-	-	-
4	**<0.0001**	NS	NS	-	-	-	-	-	-
5	**<0.0001**	NS	NS	NS	-	-	-	-	-
6	**<0.0001**	NS	NS	NS	NS	-	-	-	-
7	**<0.0001**	NS	NS	NS	NS	NS	-	-	-
8	**<0.0001**	NS	NS	NS	NS	NS	NS	-	-
9	**<0.0001**	NS	NS	NS	NS	NS	NS	NS	-
10	**<0.0001**	**0.0014**	NS	NS	NS	NS	NS	NS	NS
Rank normalized amplitudes (EMG_{sonic})	1	2	3	4	5	6	7	8	9
2	**0.0003**	-	-	-	-	-	-	-	-
3	**<0.0001**	NS	-	-	-	-	-	-	-
4	**<0.0001**	NS	NS	-	-	-	-	-	-
5	**<0.0001**	NS	NS	NS	-	-	-	-	-
6	**<0.0001**	NS	NS	NS	NS	-	-	-	-
7	**<0.0001**	NS	NS	NS	NS	NS	-	-	-
8	**<0.0001**	NS	NS	NS	NS	NS	NS	-	-
9	**<0.0001**	NS	NS	NS	NS	NS	NS	NS	-
10	**<0.0001**	NS	NS	NS	NS	NS	NS	NS	NS
Rank latencies	1	2	3	4	5	6	7	8	9
2	**<0.0001**	-	-	-	-	-	-	-	-
3	**0.0004**	NS	-	-	-	-	-	-	-
4	**<0.0001**	NS	NS	-	-	-	-	-	-
5	**<0.0001**	NS	NS	NS	-	-	-	-	-
6	**<0.0001**	NS	NS	NS	NS	-	-	-	-
7	**<0.0001**	NS	NS	NS	NS	NS	-	-	-
8	**<0.0001**	NS	NS	NS	NS	NS	NS	-	-
9	**<0.0001**	NS	NS	NS	NS	NS	NS	NS	-
10	**<0.0001**	NS	NS	NS	NS	NS	NS	NS	NS

NS, Non-Significant differences. Values in bold indicate significant differences. Results refer to the Dunn's multiple comparison tests. Significance level was determined at $P<0.05$.
